# Interactions of Host Proteins with the Murine Leukemia Virus Integrase

**DOI:** 10.3390/v2051110

**Published:** 2010-05-05

**Authors:** Barbara Studamire, Stephen P. Goff

**Affiliations:** 1 Brooklyn College of the City University of New York, Department of Biology, 2900 Bedford Avenue, Brooklyn, NY 11210, USA; E-Mail: bstudamire@brooklyn.cuny.edu; 2 Columbia University College of Physicians and Surgeons, Department of Biochemistry and Molecular Biophysics and Howard Hughes Medical Institute, 701 West 168 Street, New York, NY 10028, USA

**Keywords:** retroviruses, integrase, MoMLV

## Abstract

Retroviral infections cause a variety of cancers in animals and a number of diverse diseases in humans such as leukemia and acquired immune deficiency syndrome. Productive and efficient proviral integration is critical for retroviral function and is the key step in establishing a stable and productive infection, as well as the mechanism by which host genes are activated in leukemogenesis. Host factors are widely anticipated to be involved in all stages of the retroviral life cycle, and the identification of integrase interacting factors has the potential to increase our understanding of mechanisms by which the incoming virus might appropriate cellular proteins to target and capture host DNA sequences. Identification of MoMLV integrase interacting host factors may be key to designing efficient and benign retroviral-based gene therapy vectors; key to understanding the basic mechanism of integration; and key in designing efficient integrase inhibitors. In this review, we discuss current progress in the field of MoMLV integrase interacting proteins and possible roles for these proteins in integration.

## 1. Introduction

The development of safe, effective retroviral-mediated gene delivery vectors has been a crucial aspect of gene therapy, their promise offering hope of a cure to patients suffering from genetic diseases. Retroviruses have a distinct advantage as delivery vehicles over transient viral or episomal vectors in that integration of the transferred gene into the host cell chromosome is permanent. However, the unique ability of retroviruses to mediate stable integration is not without cost. Possible side effects and adverse outcomes are insertion of the transgene in the vicinity of an oncogene, thus deregulating it; inactivation of genes by insertional mutagenesis; silencing of the transgene; or chronic induction of immunological reactions.

Identification and analysis of the roles of host factors that may mediate the selection of proviral insertion sites will aid in our understanding of this phenomenon, ideally allowing modification of host-viral interactions to prevent adverse outcomes. The information obtained from these studies, in combination with the careful engineering and design of retroviral vectors should improve the outcome of gene transfer as a tool in therapeutic technology. In addition to addressing problems with gene therapy vectors and the sites of viral integration, identification of interacting host factors may advance our understanding of the means by which retroviruses co-opt these factors and use them to direct integration.

Efficient retroviral gene expression and propagation depends on the stable integration of a double-stranded DNA viral genome into the genome of the target cell. The key protein mediating this essential event is the viral integrase. Three structural domains are conserved between retrotransposon and retroviral integrases: all possess an N-terminal zinc finger motif that coordinates a zinc molecule and participates in multimerization of integrase monomers; an internal catalytic domain known as the D,D(35)E motif, and a less conserved C-terminal region [1,2]. Upon entry of the virion into the cytoplasm, the enzyme reverse transcriptase generates a linear double-stranded DNA molecule from the single stranded viral RNA genome, in the context of a large structure termed the reverse transcriptase complex (RTC). The completed viral cDNA and integrase then enter the nucleus as part of another large nucleoprotein complex, termed the preintegration complex (PIC) [3]. The extent and totality of host factors interacting with the PIC and acting in the early steps of integration remain elusive. The viral proteins capsid (CA), p12 and nucleocapsid (NC) are associated with the MoMLV PIC, but only NC and IN have been found in the nucleus; MA and CA remain in the cytoplasm [4]. Phosphorylated matrix (MA), reverse transcriptase (RT) and possibly Vpr have been reported as associated with the HIV-1 RTC or the PIC, although it remains controversial as to whether viral proteins other than integrase have roles beyond PIC association, and the functions that might be ascribed to the other viral proteins remain unclear [5–8]. The MoMLV PIC appears to enter the nucleus only during mitosis. This may be a reflection of a requirement for nuclear membrane disruption, and perhaps reflect the role of capsid as a restriction and rate-limiting factor for nuclear entry and may thus act as an integration factor in specific cell types [8–12]. HIV-1 can infect non-dividing cells as well as actively cycling cells, yet the mechanism by which this occurs remains unclear. Mutations in HIV-1 capsid impair infection in non-dividing cells, and chimeric HIV-1 virions in which CA and MA were replaced with p12, MA and CA from MoMLV demonstrated that CA is indeed the determinant for the inability of MoMLV to infect non-dividing cells [13,14]. The dependence of MoMLV on mitosis for infection also limits its range in gene transfer, but as HIV-1 is not impeded by this barrier, non-dividing cells are as susceptible to infection as are mitotic cells [15]. The compendium of these findings raise several questions: 1) Is there an as yet undefined downstream function for CA in MoMLV integration, given that it is the determinant for infection in specific mouse strains? 2) Should cofactor interactions with MoMLV CA and IN be examined simultaneously? 3) Why is capsid dispensable for HIV-1 nuclear entry? Is this an apparent evolutionary advantage acquired by HIV-1? If so, why has MoMLV not evolved to use a similar mechanism? Is this difference due to the limits of available cofactors, adaptation to cofactors, or is it primarily influenced by the different integration target site preferences for each virus? 4) Do cofactors act as secondary determinants for MoMLV nuclear entry and integration? 5) How is the MLV PIC retained in the nucleus following mitosis? The relative sizes of the MoMLV (160S, 30 nm) and HIV-1 (80-320S, 28 nm) PICs [16,17] do not suggest obvious answers to these questions. Host factors known to be associated with PICs include barrier-to-autointegration factor (BAF), lamina-associated polypeptide 2 alpha (LAP2 alpha), emerin, high mobility group protein HMGA1 (formerly HMG I(Y)), thyroid autoantigen Ku, and lens-epithelium-derived growth factor (LEDGF/p75), although a severe impact on viral replication has only been demonstrated upon depletion of LEDGF with respect to lentiviruses such as HIV-1 [18–24]. The lack of characterized MLV integration cofactors leaves open many questions with respect to how and when they might function to accomplish integration.

Following entry into the cytoplasm and DNA synthesis, the linear viral DNA ends are processed by integrase, which typically removes two terminal nucleotides at the 3′ ends of the long terminal repeats (LTRs). The number of nucleotides removed may vary between viruses (HIV-2 IN removes three, although this is a rare exception), as does whether or not both LTR ends are processed (*i.e.*, foamy virus) [25,26]. Moloney murine leukemia virus produces recessed 3′ OH termini with the release of a dinucleotide at each end of the LTR [27–29]. The subsequent steps of integration have been well characterized *in vitro*: generally, the two free 3′-OH linear viral DNA ends, in a concerted nucleophilic attack on the host DNA, covalently join the viral and host DNA strands, leaving a gapped intermediate with free 5′-phosphodiester viral DNA ends which are repaired by host enzymes (Figure 1) [28,30]. Although the basic retroviral integration mechanism is clear, it has been challenging to identify and characterize those cofactors that will exhibit both a significant influence on viral integration events, as well as an effect on targeting. Furthermore, those few factors that have both direct interactions with integrase and exhibit effects on viral replication are primarily effective on lentiviruses [31,32]. Thus, for the simple retroviruses such as MoMLV, proteins playing equivalent roles in integration remain even more elusive. In addition, significant questions remain regarding the influence of host proteins on import of the PIC, on the selection of target sites, on the repair of integration intermediates, and on proteins affecting the efficiency of integration.

In this review we survey the current status of host proteins interacting directly with MoMLV integrase and discuss these factors in the context of MoMLV as a viable gene therapy vector. We discuss confirmed HIV-1 integrase host factors and those implicated as direct interactors with MoMLV integrase, of which none have yet been confirmed to affect integration or replication.

## 2. Retrotransposon and phage integration

Nearly twenty years ago, elegant studies laid the groundwork for the idea that in mammalian and avian retroviral systems, the selection of integration sites might be non-random with respect to the structure of the chromatin target and examined the possibility that DNA sequence might exert an influence [33–39]. Recent findings provide evidence that host proteins are intimately involved in integration and are likely to play a prominent role in target site selection. The mechanism of Ty element integration in yeasts has served as both model and inspiration for investigating the role of host factors in retroviral integration. Such functions have been elegantly demonstrated for yeast retrotransposons Ty1, Ty3 and Ty5 [40–42]. Ty1, a copia-like element, frequently integrates within 750-bp of the 5′ end of tRNA genes, and deletion of the RecQ helicase *SGS1* leads to an increase in multimerization of the Ty1 genome and to the transposition of heterogeneous Ty1 multimers, suggesting a prominent role for this cofactor [43]. In the case of Ty3, targeting to within one or two nucleotides of tRNA gene transcription start sites *in vivo* is suggested to be mediated by an interaction with TFIIIB and TFIIIC [44,45]. Mutations in Sir4p that disrupt telomeric silencing lead to a loss of Ty5 targeting to heterochromatic regions, indicating that targeting is controlled by transcriptional modifiers [46]. Ty5 integration can also be redirected by replacement of the integrase targeting domain with peptide motifs known to bind to other proteins: the result is a redirection of Ty5 to sites bound by these proteins [47]. These elegant studies demonstrate that retroelement target site selection can be manipulated and provide useful models for potential redirection of retroviral vectors in gene therapy. This has also been demonstrated in recent studies with an HIV-1 cofactor (see section 3 below).

The LTR retrotransposons and retroviruses share two highly conserved regions: the N-terminal zinc-binding HHCC subdomain and the D,D(35)E motif. The HHCC domain is less conserved than the central catalytic D,D(35)E motif. We were unable to locate literature noting precise degrees of conservation between retroviral integrases and those of the retrotransposons, likely due to sequence divergence between these elements. However, Khan *et al*. found that in the D,D(35)E motif there are regions in which 75% of 16 amino acid substitutions are conservative, and in another set of 16 residues, 50% of the substitutions are conservative [48]. A much less conserved region within the C-terminal subdomain known as the GPY/F module (G-(D/E)-X_10–20_) has been defined in a subset of members of the Ty3/Gypsy group and in a limited number of retroviruses [49]. This region is proposed to be necessary for the integration specificities of these elements. MoMLV and Baboon endogenous retrovirus (BaEV) are among the retroviruses possessing the GPY/F module [49].

There is little sequence similarity between the integrases of retroviruses and those of bacteriophages, however, there is a low degree of conservation between the catalytic domains of Mu transposase and retroviral integrases (15%). These enzymes belong to a superfamily of polynucleotidyl transferases [50]. Phage lambda integration in bacteria is site-specific, rather more analogous to the integration of retrotransposons in yeast, than to the patterns observed for retroviral integration in mammalian cells. However, the use of cofactors by phage, and in particular, the interactions between the *E. coli* phages lambda and Mu with integration host factor (IHF) and HU support a model of usage of common cofactors by more than one virus. The IHF protein is a histone-like heterodimeric DNA-binding protein that was originally identified as an accessory protein in lambda integration and has roles in *E. coli* gene expression, replication, transcription, and translation. Lambda uses IHF for both integration and excision of its genome in the lysogenic cycle, in conjunction with other phage encoded proteins [51]. In the integration reaction, IHF bends DNA by binding to lambda *attP* sites, bringing the Integrase (Int) into proximity of the phage core DNA sites, thus facilitating the reaction [52]. IHF and HU are present in many species of bacteria, and IHF is structurally related to HU. HU also bends DNA, and although it is a non-specific DNA binding protein, it can substitute for IHF in both the integration and excision reactions. HMG1 and HMG2 can also replace IHF in integration and excision, and so too can the core histone dimer H2A-H2B to some extent, although not in excision [51]. Bacteriophage Mu can also utilize HU or IHF in the strand transfer reaction [53]. The organization of the Mu transpososome, the complex of DNA and transposase, can serve as a general model for the organization of retroviral PICs [50,54,55].

## 3. Identifying and isolating interacting factors

The literature is fairly barren with respect to *in vivo* data for direct host factor-MoMLV IN interactions. This places limitations on our ability to discuss this area of research, yet we hope the data will soon be forthcoming. In this section we discuss methods used to identify, isolate and characterize cofactors for other viral proteins as well as those for integrase. HIV-1 is the most intensely studied retrovirus, so the majority of these cofactors have been identified in the context of this virus. Opportunities are many in which the viral integrase may interact with host factors during the early stages of infection. Theoretically, these factors may interact with the RTC, with the PIC either before or after nuclear entry, during integration, and after integration during gap repair. Separating the nature of these interactions into discrete steps is a challenge, but clues to potential functions in integration may sometimes be obtained from the identity of the cofactor in obvious cases. A fairly obvious example would be the potential role of DNA repair proteins in gap repair. Less obvious examples would be the roles of transcription factors, stress response proteins, and RNA binding proteins. Also, those factors identified as playing roles in integration, but with no demonstrable direct interaction with IN have less obvious, but nonetheless, sometimes significant roles. The conclusion from the accumulated data being that broad applications such as genome-wide screens will probably help to narrow the list of potential cofactors. A recent meta-analysis of nine genome-wide studies for overlapping HIV-1 interacting factors suggests that factors identified in multiple screens are likely to be bona fide interactors (see references contained therein) [56]. Some of these factors may also be developed as attractive targets for drug development. Integrase appears to be a major determinant for target selection, as demonstrated by swaps of MoMLV Gag and IN proteins in HIV-1 virions. When cells were infected with chimeric viruses comprised of MLV IN and/or MLV Gag in the context of the HIV-1 genome, the integration patterns observed were similar to those observed for MoMLV [57]. This study also identified a role for Gag in integration *in vivo* [57]. The various classes of retroviruses appear to make distinctions in target site selection and their preferences may be influenced by host factors specific to the virus and the cell type. Genome-wide screens of integration sites and transcription profiling of infected cells have contributed an immense amount of information to our understanding of preferred targets for integration [58]. Studies analyzing hundreds of integration sites seem to finally settle the question of whether or not retroviral integration is a completely random process, at least for some retroviruses. Wu *et al.* performed a genome-wide screen of pseudotyped MoMLV proviruses in HeLa cells that identified integration target preferences as those surrounding the transcription start sites of genes and near CpG islands [59]. The same study also analyzed wild type and pseudotyped HIV-1 in human H9 cells and HeLa cells, respectively, and found that HIV-1 integration was favored in the transcribed regions of genes [59]. Schröder *et al*. found that integration of HIV-1 in Sup T1 cells occurred preferentially into transcription units with regional hotspots and all of the targeted genes were predicted to be transcribed by RNA polymerase II, with no bias observed for integration at transcription start sites [60]. A recent study found that MLV integrase and its LTR U3 region were the major determinants for transcription factor binding site integration events [61]. This study has important ramifications for directing and retargeting retroviral vectors for use in gene therapy, analogous to the studies redirecting Ty5 by replacement of the integrase-targeting domain.

Avian sarcoma-leukosis virus (ASLV) infections in 293T-TVA cells (293T cells engineered to express the receptor for ASLV) displayed no preference for integration near transcription start sites and only a weak bias towards active genes [62]. A genome-wide study of avian sarcoma virus (ASV) found no preference for integration into highly expressed genes, but did identify a preference for RNA polymerase II transcription units in HeLa cells [63].

When isolated from their natural context, integrase proteins have proven themselves to be notoriously insoluble, making them difficult to manipulate *in vitro*. Finding methods around these limitations has allowed researchers to identify a limited number of HIV, ASV and MLV integrase-interacting host factors. The techniques of PIC component analysis, co-immunoprecipitation with integrase and the yeast two-hybrid system have been central in this effort. The yeast two-hybrid method, albeit not without its own limitations, has been used frequently to identify integrase and other retroviral protein/cofactor interactions, and to study interactions between known protein binding partners. The major drawback in all screens is that the candidate proteins identified must be extensively verified by functional assays.

Yeast two-hybrid analysis [64] was used to identify host proteins that interact with MoMLV RT protein (eRF1) [65]; avian sarcoma virus IN (Daxx) [66]; HIV-1 Gag protein (Cyclophilins A and B) [67]; HIV-1 IN protein (INI1) [68], Gemin2 [69] and recently, transportin SR2, (TRN-SR2) [70]. Daxx was recently found to repress HIV-1 gene expression via recruitment of HDACs through its interaction with IN [71]. HIV-1 integrase was used as the bait to screen an human cDNA library using the yeast two-hybrid system, resulting in the identification of Integrase interactor 1 (INI1), a SNF5 homologue. This protein was the first protein identified to interact directly with HIV-1 integrase [68]. While the initial studies demonstrated that INI1 stimulated DNA joining reaction *in vitro*, recent studies indicate that INI1 is incorporated into virions, is required for efficient particle production [72], and stimulates Tat-mediated transcription of HIV-1 RNA [73]. A recent report demonstrated a direct interaction between HIV-1 IN, INI1 and SAP18, a component of the histone deacetylase complex Sin3a-HDAC1 [74]. The interaction occurs in the absence of the SWI/SNF core components BRG1, BRM, BAF155, and BAF170, and a Sin3a-HDAC1 complex is selectively incorporated into HIV-1 virions. Both INI1 and the Sin3a-HDAC1 components interact with HIV-1 IN, but not with SIV IN and are not incorporated into the SIV virions. The authors suggest that IN may recruit Sin3a-HDAC1 via interactions with INI1 and SAP18 to influence RT function, as active an Sin3A-HDAC1 complex appears to be required for early reverse transcription [74]. To date, no direct effect on targeting has been documented for INI1. No comparable activity has been identified for MoMLV, and it is possible that these activities may be virus- or cell type-specific.

The first cofactor with a defined, specific and integral role in HIV-1 integration, Human lens epithelium-derived growth factor (LEDGF), was identified both by its association with exogenously expressed HIV-I IN [75,76] and in a yeast two-hybrid screen [77]. Years of analysis has defined what are thus far unique roles for LEDGF/p75 in important aspects: in infected cells, a role in nuclear targeting of HIV-1 integrase [78] and what appears to be an essential role in HIV-1 integration and viral replication [24,79]. LEDGF/p75 would thus appear to play a major role in the integration of HIV-1 and other lentiviruses (but not all retroviridae), and is the first cofactor for which evidence of a direct role in integration has been conclusively demonstrated [32,80–82]. Alignment of the protein sequences of the mouse and human LEDGF/p75 proteins show that the proteins share an overall identity of 92% and the Cherepanov *et al.* identified integrase-binding domain of LEDGF/p75 shares 100% consensus with the corresponding region in mLedgf. However, the murine protein plays no apparent role in MLV integration, suggesting the possibility that different host factors may be required for tethering either the PIC or viral DNA to selected regions of chromatin for this virus [83,84]. In the absence of the mouse protein (in mLedgf knockout mouse embryonic fibroblasts), HIV-1 lost its ability to target transcription units, and instead exhibited an integration pattern more related to that observed for MLV [82]. This may be the most conclusive evidence that LEDGF plays a major role in targeting integration events to particular genomic regions for HIV-1, and highlights the fact that the murine proteins responsible for targeting MLV have yet to be identified. This may also be a reflection of different nuclear entry mechanisms used by HIV-1 *versus* MoMLV. The observation that MLV does not use LEDGF and that HIV integration site preferences are altered in the absence of this cofactor in mouse cells suggests that in the absence of LEDGF, HIV-1 will make use of available cofactors. The overall efficiency of integration was reduced in the Marshall *et al*. study in both mouse and human cells depleted of LEDGF/p75 [32], so it appears reasonable to conclude that local factors are less efficient substitutes. As we have no data on the influence of cofactors on MLV integration targeting, we cannot speculate on their possible use by HIV-1 in the absence of LEDGF. There is no evidence to support a ‘default’ set of cofactors, and it is possible that an incoming virus, according to its genera, avails itself of the factors present in the host. However, a considered answer to this question awaits experimental evidence.

As shown for Ty5 [47], altering the DNA binding domain of an integrase tethering, targeting host factor might successfully redirect integration events. A recent study employed replacement of the LEDGF chromatin-binding domain with chromobox protein homolog 1 (CBX1), an homolog of the *Drosophila* HP1 protein [85]. CBX1 is a non-histone heterochromatin protein that binds di- and tri-methylated histone H3K9 tails. In LEDGF knockdown cells transduced with HIV, the LEDGF/CBX1 chimera shifted integration events from those observed within RefSeq genes (in knockdown cells back complemented with LEDGF), to heterochromatic sites. The transduced virus continued expression over a two-week period, despite integration into heterochromatic regions [85].

Rad18, a DNA post-replication repair protein (translesion repair) that heterodimerizes with the ubiquitin-conjugating enzyme Rad6, was examined as an HIV-1 IN cofactor [86]. The protein was found to stabilize IN against degradation in HEK 293T cells, and to suppress viral infection [86,87]. Other DNA repair enzymes, ATR, ATM, DNA-PK_cs_, XRCC4, and Ku80 have also been proposed to affect the efficiency of integration or the integrity of integration products for ASV, HIV-1 or MoMLV [88,89]. The results of these studies suggest that DNA-PK acts in repair of integration intermediates as DNA integration was reduced in *scid* pre-B cells deficient for this enzyme [89]. However, it is unknown whether or not these proteins are essential for integration [90–92]. A recent study examined the effects of ATM, DNA-PKcs, NBS1 and Mre11 on HIV-1 and MoMLV transduction and found a significant defect only in cells lacking DNA-PK_cs_ [93]. The same study identified altered host/viral DNA junctions consisting of insertions/deletions (IDLs) in cells deficient for ATM, NBS1 and Mre11 [93]. This interesting study suggests that the selection of different parameters for measurement might yield quite subtle results. Might DNA mismatch repair exert a similar subtle effect in the assay employed by Sakurai *et al.*, as IDLs are the primary target of this repair pathway? It is worth noting that a previous study employing Msh2 deletion cells found no decrease in GFP-virus transduction, but the junctions between viral and host sequences were not examined [94]. The variance in results between these studies may be a result of the redundancy in mammalian DNA repair enzymes, as well as the choice of events and types of activities measured.

Among the techniques that can be used to verify the interactions obtained from yeast two-hybrid screens is RNA interference. It may be employed *de novo* to identify an effect on viral replication when expression of the factor of interest is suppressed, or it may be used to determine the functional activity of a candidate protein on the virus under study. Transportin-SR-2 (TRN-SR2) was identified from a genome-wide screen of factors inhibiting HIV-1 infection [95]. TRN-SR2 and TRN-SR1 are alternatively spliced versions of the gene encoded by *TNPO3* [96]. TRN-SR2 is a karyopherin that transports serine/arginine-rich (SR) proteins into the nucleus. Among this class of proteins, importin alpha/importin beta are proposed to be important for retrovirus infection [97,98]. TRN-SR2 was also identified in an HIV-1 IN yeast two-hybrid screen and experimental results demonstrate that depletion of this factor by RNAi results in the disappearance of PICs in the nucleus after acute infection, suggesting that this protein participates in the import of the PIC into the nucleus [70]. A recent study of TRN-SR2 (TNPO3) knockdown cells revealed a significant reduction in viral titers for simian immunodeficiency virus (SIV) and HIV-1, slight reductions for equine infectious anemia virus and bovine immunodeficiency virus (EIAV and BIV), and essentially little or no effect on titers for MLV, Rous sarcoma virus (RSV) and feline immunodeficiency virus (FIV) [99]. However, in the same studies, pull-down assays between the various integrases and TRN-SR2 revealed that although MLV and FIV IN bound TRN-SR2, these binding affinities did not correlate to the observed lack of an effect on infection. Additional analyses in stable knockdown cells using chimeric MLV/HIV-1 viruses in which MLV MA-p12-CA replaced HIV-1 MA-CA, revealed that infection levels were dependent on the presence of HIV-1 CA, implicating capsid, not IN, as the determinant for sensitivity to TRN-SR2 [99]. Importin 7 (Imp7) has been proposed to play a role in nuclear import of HIV-1 DNA as well, and knockdown of Imp7 reduced nuclear accumulation of HIV-1 DNA, but had only modest effects on replication [100]. TRAF and TNF receptor-associated protein (TTRAP), a promyelocytic leukemia nuclear body associated protein (PML-NB), was isolated in a yeast two-hybrid screen with HIV-1 integrase as bait and its knockdown was shown to reduce viral integration [101]. A recent yeast two-hybrid screen using acetylated HIV-1 IN as bait identified thirteen cofactors [102]. HIV-1 IN was tethered to the histone acetyltransferase catalytic domain of p300 and used to screen a T-cell GAL4 activation domain cDNA library. Among the clones isolated in the screen were LEDGF/p75, high-mobility group nucleosomal binding domain 2 (HMGN2), elongation factor 1 alpha 1 (eIF1A-1), basic transcription factor 3 isoform b (BTF3b), Exportin 2 (Exp2), Kruppel-associated protein 1 (KAP1), Ran-binding protein 9 (RanBP9) and many clones of eukaryotic translation initiation factor subunit H (eIF3h) [102].

It may be the case that several of the identified factors act coordinately or at discrete steps in HIV-1 nuclear import and integration, and further research may yet uncover global mechanisms for early integration steps. Factors specific for each virus may account for the differences between the integration specificities observed.

It is possible to biochemically isolate preintegration complexes from infected cells and to identify interacting host factors by analysis of their components. Host factors associated with PICs can be identified by purification from salt-stripped PICs isolated from cell lysates. Cell lysates should be analyzed for restoration or complementation of integration activity *in vitro*, and direct interactions with integrase will require confirmation by other methods. Proteins that were identified using these methods include HMG I(Y), now called HMGA1, and barrier to autointegration factor (BAF) [103,104]. Transcriptional profiling may be used to identify proteins whose expression is up- or downregulated upon viral infection. This method will provide a large number of potential target proteins for further investigation, although specific interactions with integrase will not be revealed by this method [105–108].

## 4. Why Study MoMLV Integrase?

MoMLV, a gammaretrovirus, is classified as a “simple” retrovirus, while lentiviruses such as HIV-1 are classified as “complex” retroviruses. The simple retroviruses encode Gag, Pro, Pol, and Env, whereas the complex viruses encode these proteins as well as several accessory proteins having a range of functions [109]. Because of its simplicity, MoMLV is often used as the model virus to study the basic retroviral life cycle. Although the results obtained from studying MoMLV cannot always be generalized to all retroviruses, this model allows researchers to address basic questions with respect to viral infection: among these are the use of membrane receptors, cell cycle control, viral gene expression, host-virus interactions, and viral transport. MoMLV has been the most frequently used vector for gene transfer, but its status as an oncoretrovirus makes its continued use in this capacity controversial and problematic. Identification and characterization of host factors that may modulate integration for this virus are critical to ameliorating or altering its target site selection. Currently, there is no biochemical model for MoMLV integration that includes its use of host factors.

Recent findings on the possible involvement of xenotropic murine leukemia virus-related virus (XMRV) in prostate cancer suggest that synergism between viruses may lead to the production of infectious viruses, adding another potential complication to the use of MoMLV as a gene therapy vector [110,111]. It might be useful to examine interactions between XMRV integrase and the putative cofactors identified for MoMLV integrase to determine if any of the MLV interactions are recapitulated.

## 5. MoMLV Integrase Interacting Factors

Until recently, there was no available data on proteins that interact physically with MoMLV IN. A recent screen identifying 27 putative MoMLV integrase cofactors suggests that for this virus, components from multiple host pathways may be involved at different stages in the integration pathway. The proteins identified in the MoMLV screen along with proteins isolated by other means (Emerin, BAF and Lap2 alpha) (Table 1) can be categorized into the following three broad groups. (I) DNA binding proteins: chromatin binding, DNA repair, and transcription factors; (II) RNA binding proteins: representing several components of the spliceosome; and (III) miscellaneous factors that include stress-response and transport proteins [112]. Identification of functional roles for these factors and determination of specific, if any, effects on viral infectivity and replication await confirmation by *in vivo* studies, but it is encouraging that there is now a group of potential cofactors available for analysis and that some of these factors have been identified elsewhere by other methods: Ddx5 and SF3B2 [56]. With respect to the thirteen proteins in the DNA binding group, although they share no simple sequence similarity, each may contain a common IN recognition feature. A degree of support for this idea is provided by the results of limited yeast two-hybrid assays in which truncated IN proteins were tested with a small group of the potential cofactors. The yeast two-hybrid studies identified interactions between a D,D(35)E /C-terminal fusion of IN and six of the putative cofactors: B-activating transcription factor (B-ATF), acute lymphocytic leukemia gene 1 fused from chromosome 9 (AF9), bromodomain-containing protein 2 (Brd2), enhancer of zeste E(z) homolog 1 (ENX-1/EZH2), and TATA binding protein ABT1 (ABT1) [112]. This suggests that the C-terminus of MoMLV IN may have extensive interactions with host factors. A thorough analysis of possible roles for these factors in integration may help address problems with MoMLV-based gene therapy vectors, and may provide tools enabling scientists to address the issue of whether or not cofactors are involved in the nuclear import of the MoMLV PIC. Below, we will summarize some of the recently identified MoMLV integrase cofactors and our current understanding and expectations for their role in virus replication.

Polycomb group (PcG) proteins act not only as transcriptional repressors during the regulation of developmental genes, but also have dynamic roles in other biological processes, such as X-inactivation, actin polymerization, cell cycle control, cancer development, and genomic imprinting [113]. ENX-1/EZH2 is one of the two mammalian enhancer of zeste homologs, and in *Drosophila*, mutations in PcG genes lead to homeotic transformations due to a failure to maintain appropriate gene expression. In mammals, the core components of the Polycomb Repressive Complex 2 (PRC2) are ENX-1/EZH2, the extra sex combs homolog embryonic ectodermal development factor (EED), and the suppressor of zeste homolog (SUZ12) [114,115]. One current model suggests that maintenance, but not initiation of transcriptional silencing and modulation of chromatin structure is effected by PRC2 via histone methylation, and that Polycomb Repressive Complex 1 (PRC1) is recruited to the Polycomb Response Element (PRE) by PRC2 epigenetic marks [116]. However, PREs have not been defined in mammalian cells. Interaction of the complexes with the RNA interference machinery is also implicated in silencing [117]. EZH1 and EZH2 are histone methyltransferases that catalyze the addition of methyl groups to histone H3 at lysine 27 via their SET domains, a function that recruits other Polycomb group proteins to repress chromatin [118]. In association with different isoforms of EED, the methylation specificity of EZH2 can be altered to target histone H1 lysine 26 as well [119]. Overexpression of EZH2 is implicated in a wide array of diseases, most frequently breast and prostate cancer. Invasive cell growth requires the EZH2 SET domain and multiple myeloma (MM) cells induce ENX-1/EZH2 transcript expression *in vivo* and these cells are growth arrested by siRNA against EZH2 [119–121]. An alternative PRC2 complex containing the other mammalian E(z) homolog, EZH1, can methylate H3K27 in the absence of EZH2, although it cannot fully complement EZH2 activities [122].

The first PcG protein identified as a putative retroviral cofactor was EED, which was isolated in conjunction with three HIV-1 proteins. A yeast two-hybrid screen using HIV-1 Matrix as bait subsequently demonstrated that EED also interacted with HIV-1 IN [123,124]. The EED/HIV-1 IN interaction produced an increase in integration *in vitro*, and the IN C-terminus was required for this interaction [124]. A separate Nef yeast two-hybrid screen also recovered EED, and Nef was later shown to simulate an integrin receptor signal, translocating EED from the nucleus and relocalizing it to the plasma membrane. This activity also produced an increase in Tat-mediated transcription [125]. A study by Rakotobe *et al.* found that EED exerts a negative effect on HIV-1 replication, mediated by Nef [126]. These studies have not yet investigated possible interactions between EZH1, EED, SUZ12, and ENX-1/EZH2 with HIV-1 or with MoMLV IN, but the identification of the two proteins by different groups hints at a major role for the PRC2 complex in the viral life cycle. The degree of complexity with respect to the layers of organization, interactions and activities of the PcG proteins and chromatin, combined with a layer of viral interactions suggests that a complete picture of the viral-PRC2 interactions may be difficult to uncover. It is tempting to speculate that EZH2 and EED may play equivalent roles in the life cycles of both HIV-1 and MoMLV: the two proteins may interact with one another, or exhibit distinct roles dependent on the composition of different PcG complexes, which may be cell-type or virus-specific. The simplest models for PcG activity propose that PcG proteins counteract the effects of the trithorax group of transcriptional activators (TrxG), but both are required to maintain appropriate expression of their target genes. There appear to be overlapping binding affinities for TrxG and PcG complexes to PREs and to Trithorax Response Elements (TREs), as well as binding to other proteins involved in activation or silencing [127]. The early models of distinct complexes with separable functions have been complicated by the identification of multiple isoforms forming alternative complexes with overlapping binding affinities and functional activities [128].

The mixed lineage leukemia translocated to chromosome 3 homolog (*Mllt3*), also called acute lymphocytic leukemia gene 1 fused from chromosome 9 (*AF9*), is often found in reciprocal translocations with the mixed lineage leukemia gene (*MLL*) in acute myeloid leukemia (AML). MLL is the mammalian trithorax homolog, and in mice, is required for embryogenesis and binds to promoter sequences in Hox gene clusters, suggesting it may regulate these genes [129]. Mice nullizygous for *af9* have homeotic transformations and are perinatal lethals, in support of AF9 as a *Hox* gene master regulator [130]. AF9 has also been proposed as a transcriptional activator because it contains a nuclear localization signal and a serine/proline-rich domain that is routinely retained in MLL-AF9 fusions. Although AF9 function has yet to be defined, it is a YEATS family member; an nGAP homolog; has a pleckstrin homology (PH) domain; contains a Gap–related domain (GRD); and is a RAS GTPase activating protein [131]. The mouse and human homologs of the *Drosophila* Polycomb group protein Pc3 and the BCL6 corepressor, BcoR, both transcriptional repressors, interact with the C-terminus of AF9 [132,133].

The Ku autoantigen was first isolated from patients exhibiting polymyositis-scleroderma overlap syndrome, and its name was derived from the first two letters of one patient’s name [134]. The 70 kD subunit of the Ku70/Ku80 heterodimer (Ku86), Ku70/XRCC6, was identified by isolation of an antibody from patients with autoimmune thyroid disease and lupus erythematosus, and these antibodies were later used to isolate cDNA clones encoding p70 [135]. The Ku86 heterodimer is proposed to be the first protein recruited to DNA double-strand breaks and acts as the damage sensor. It is an ATP-dependent DNA helicase that functions as a sliding clamp and recruits DNA-PK_cs_, DNA polymerases, and ligases to the site of DNA damage in a mechanism similar to that used by the sliding clamp PCNA [136]. The Ku86 heterodimer functions in the repair pathway of non-homologous end joining (NHEJ), binding to the free ends of linear double-stranded DNA, hairpin DNA, and 5′ or 3′ overhangs [137]. The heterodimer also functions in V(D)J recombination [138]. Ku70, and by extension Ku80, act with Telomere repeat factor 2 (TRF2) to suppress homologous exchange of telomeres between sister chromatids [139]. The NHEJ complex functions in the repair of DSBs generated in Ty1 retrotransposition [140,141] and in retroviral integration [23,89,92,142]. Indeed, the mechanism of V(D)J recombination is similar to that of retroviral integration [143]. The isolation of Ku70 supports previous findings of NHEJ protein involvement in the repair of retroviral integration intermediates and possibly in the formation of one- and two-LTR circles for MLV, HIV-1 and other retroviruses.

The Rad two homolog-1 (RTH1 or Rad27), also known as Flap endonuclease-1 (Fen1), is a structure-specific 5′ metallonuclease that functions in the maintenance of genome stability. The functions of Fen-1 include apoptotic DNA fragmentation, resolution of Okazaki fragments in lagging-strand DNA synthesis, long-patch base excision repair, and NHEJ [144]. In yeast cells, deletion of Rad27 results in an increased rate of recombination and an increased rate of chromosome loss, indicating its significance in genomic stability [144]. The transcription coactivator p300 acetylates Fen-1 [145], and both proteins have been implicated in retroviral integration [146]. Fen-1 also interacts with proliferating cell nuclear antigen (PCNA), replication protein A (RPA), hnRNP, and with DNA polymerases beta, alpha, and epsilon [147].

Bromodomain motif-containing proteins are a large family of proteins that are typically implicated in transcriptional activation, chromatin repression and epigenetics [148]. The bromodomain derives its name from the *Drosophila* brahma protein in which the motif was initially identified, and a majority of histone acetyltransferases and transcriptional activators contain this motif [149]. Bromodomain-containing protein 2 (Brd2; also known as fsrg1 and RING3) is a kinase localized to the nucleus and functions as a transcriptional co-activator [150]. A Brd2 complex has been identified containing E2 promoter binding factor (E2F), histones, Brg-1, p300, Cyclin A2, HDAC11, CBP, and TAF_II_250 [148,151]. Denis *et al.* showed that overproduction of Brd2 resulted in elevated Cyclin A transcription which destabilized the cell cycle, presumably because Brd2 associated with the *cyclin A* promoter at both the G_1_ and S phases [148]. Brd2 appears to modulate viral transcription and episomal DNA replication through its interaction with the chromatin-binding domain of the Kaposi’s sarcoma-associated Herpes virus (KSHV) latency-associated nuclear antigen 1 (LANA-1) [152]. Quite possibly, Brd2 may serve as a tether for the KSHV genome to mitotic chromosomes via LANA-1 in a manner similar to that observed between Brd4 and the Bovine papillomavirus (BPV) E2 protein. Baz2b is a bromodomain family member whose function is mysterious, but the protein has been proposed to be involved in transcriptional regulation and to interact with the chromatin remodeling protein ISWI (Imitation SWI), a member of the SWI2/SNF2 family of ATPases [153].

Basic leucine zipper transcriptional factor ATF-like (B-ATF) is a member of the AP-1/ATF superfamily of transcription factors [154], whose tissue-specific expression is limited to hematopoetic tissues and cells in human and mouse [155]. B-ATF does not exhibit interactions and functions typically observed with many other ATF family members in that it does not homodimerize, it does not contain a functional transcriptional activation domain, nor does it dimerize with Fos, but instead binds Activator protein-1 (AP-1) consensus DNA sites by forming heterodimers with JunB, JunD and c-Jun [155]. Thus, B-ATF acts as a natural dominant-negative regulator of AP-1 mediated transcription, functioning as a non-activating competitor for c-Fos in the AP-1 dimer to inhibit cell growth. Evidence for this activity is exhibited in mouse cells where ectopically expressed B-ATF reduces transformation by *H-ras* and *v-fos* [155]. It is also of interest to note that in mouse, T-cell lymphoma-specific MoMLV integrations have been identified at the *Fos/Jdp2/Batf* locus [156], and that the Epstein-Barr nuclear antigen 2 (EBNA2) induces up-regulation of B-ATF in EBV infected human B-cells, leading to viral latency rather than entrance into the lytic cycle [157].

The Zinc finger protein p38 is a transcriptional activator containing seven Cys_2_His_2_ type zinc fingers, a novel N-terminal domain and a SCAN box (SRE-ZBP, CTfin51, AW-1 (znf174) Number 18) known as the leucine rich region [158]. Mammalian two-hybrid studies have identified the SCAN domain as capable of transcriptional activation, and the domain has also been identified as a protein-protein interaction motif [159,160]. Znfp38, also known as Zscan21, CTfin51, RU49, and Zipro1, is generally expressed in skin, and increased gene dosage results in hair loss, a characteristic of epithelial cell proliferation and abnormal hair follicle development [161]. Northern blotting and in situ hybridization studies show that this protein is up-regulated in testis during spermatogenesis [159] and is a marker for cerebellar granule cell precursors in developing cerebellum [161]. What these diverse functions imply about possible roles for these transcription factors in the life cycle of retroviruses, and particularly in integration remains to be defined.

Peroxisome proliferative-activated receptor gamma, coactivator-1 alpha (formerly known as PGC-1, also known as PPARGC1A), is a nuclear hormone and steroid receptor coordinating diverse organ- and cell-specific transcription programs in response to stress. PPARGC1A interacts with many transcription factors and two proteins in the family sharing domain organization have been identified: PGC-1-related coactivator (PRC) and PGC-1beta (PERC/ERRL-1) [162,163]. These proteins share an N-terminal region containing a nuclear hormone receptor interacting motif, an LXXLL coactivator motif, an RS-rich domain, and a C-terminal RNA binding motif [162,163]. The C-terminal domain of PGC-1alpha has been implicated in the splicing of target genes via its RNA binding motif [164]. PGC-1alpha and PRC interact with nuclear respiratory factor 1 (NRF-1), which activates a number of mitochondrion-related genes, linking metabolism and cellular growth [165]. For example, NRF-1 is implicated in the biosynthetic pathways of two rate-limiting enzymes in purine nucleotide biosynthesis, the CXCR4 chemokine receptor and the human poliovirus receptor CD155, by the presence of binding sites for NRF-1 in the promoters of these genes [165–167]. PRC enhances NRF-1-dependent transcription *in vitro* and *in vivo* [162]. PRC is cell cycle regulated and is broadly expressed in all tissues as evidenced by the fact that cells arrested in G_0_ exhibit extremely low levels of PRC mRNA and protein, and expression levels are returned to detectable levels when serum is added to the culture medium [162].

The ERM (Ezrin-Radixin-Moesin) family of proteins regulate cortical structure and are active in the Rho and Rac signaling pathways [168]. Between them, the ERM proteins share 75% amino acid sequence identity and all proteins share a conserved domain that binds the plasma membrane known as the band 4.1 ERM domain (the four-point one ezrin radixin moesin, or the FERM domain), comprising about 300 residues of the N-terminal region in each protein [169]. Expression of the proteins is usually cell-type and organ-specific. ERM proteins each contain a stretch of approximately 30 residues in their carboxyl-terminal domains that bind F-actin. The binding of phosphatidylinositol 4,5 bisphosphate (PIP_2_) to Radixin results in unmasking of its FERM domain, thus activating it. [170]. Phosphorylation (induced by growth-factors) at C-terminal threonines by protein kinase C-alpha, protein kinase C-theta, or by Rho-associated kinase stabilizes the now unmasked ERM proteins in an open form, thereby regulating their actin binding activity [171]. The only ERM protein so far identified as a bona fide tumor suppressor is the gene for neurofibromatosis-2 (NF-2), Merlin (moesin-ezrin-radixin-like protein) [170]. Endogenous levels of Moesin inhibit viral replication and overexpression of the protein was shown to inhibit infection of the HIV and MLV viruses prior to the initiation of reverse transcription [172]. Recently, Ezrin was found to block infection of MoMLV and HIV-1 in Rat2 cells also at a step preceding reverse transcription, and a number of other studies have identified roles for members of the ERM family in varied retroviral processes and in interactions with cell surface receptors [173–177]. Radixin is the first ERM family member to be directly implicated as a cofactor in integration and exploration of a larger role for this protein and possible interactions with the other ERM proteins in viral infection may add considerable knowledge to our understanding of global viral transport mechanisms.

Major components of the pre-mRNA splicing machinery in the eukaryotic nucleus are the small ribonucleoproteins (snRNPs). Each snRNP is composed of one to two small nuclear RNAs (snRNAs) bound to a set of seven RNA-binding proteins, called Sm proteins: SmB/B’, SmD1, SmD2, SmD3, SmDE, SmF, and SmG, which are common to all snRNPs except U6 snRNP [178]. The Sm proteins bind to a conserved uridine rich sequence, typically PuAU_4–6_GPu, on each snRNA called the Sm site. These Sm cores are then assembled into a ring on snRNAs by the SMN (Survival of Motor Neuron) complex, which is essential for this assembly [178]. *SMN* is the gene for spinal muscular atrophy (SMA) and reduction of protein expression levels produces massive cell death that results in early embryonic lethality in murine embryos, and death in humans between the ages of two to four in the most severe manifestation of the disease. Mice have only one genomic copy, whereas in humans, the gene exists as an inverted duplication on chromosome 5 [179]. The SMN protein is part of a large complex of at least six to seven Gemin proteins (Gemins 2–8) that organize and decipher the snRNP code [179–181]. SMN has been shown to interact directly with Gemins 2, 3, and 8 [178,182]. When SMN levels were reduced by an SMA-inducing mutation, the relative amounts of Gemins in the SMN complex were also decreased [182]. Hamamoto *et al.* recently used yeast two-hybrid screening to identify Gemin2 as an HIV-1 integrase cofactor [69]. In these studies, down-regulation of Gemin2 by siRNA in HIV-1 infected cells produced a block to HIV-1 infection, reduced proviral integration, reduced viral cDNA copy number, and reduced 2-LTR circle accumulation. This effect might be mediated by other Gems, some of whose levels were also reduced by knockdown of Gemin 2 and SMN [69]. Golembe *et al.* found that the seven small RNAs (HSURs) encoded by *Herpesvirus saimiri* (HVS), which are similar to host snRNPs, bind to the SMN complex and assemble the Sm cores [183]. In fact, the HSURS bound the Sm cores more strongly than did the host snRNPs, effectively out competing them [183]. These findings are interesting because the identification of SMN as a cofactor for both MoMLV and HVS supports a theory where multiple viruses may use the same host pathways. The HIV-1 IN Gemin 2 interaction also lends some support to this theory.

An essential component of the spliceosome, the U2 snRNP binds to the pre-mRNA branch site by base-pairing with the complementary RNA sequence of the U2 snRNA [184]. A complex of U2 snRNP/U1 snRNP binds to the 5′ splice site, forming a complex of U1 snRNP/U2 snRNP/pre-mRNA which in turn recruits the U4/U6, and U5 snRNPs forming an active spliceosome [184]. The now 12S U2 snRNP interacts with splicing factor 3b (SF3b) forming a pre-mature 15S U2 snRNP [185]. The 15S U2 snRNP complex binds SF3a to form a mature 17S snRNP, which interacts with nucleotides upstream of the branchpoint within the intron [185]. Splicing factor 3a subunit 3 (SF3a3; also known as Sf3a60 and Spf3a3) is a C2H2- type zinc finger protein required for assembly of the core complex and is the mammalian homolog of *S. cerevisiae* PRP9 [186]. Finally, the SF3a complex associates with the SF3b complex, the SF3b2 subunit of which interacts directly with SF3a.

The helicase dead box protein Ddx5 (Ddxp68, p68), an RNA-dependent ATPase, is a transcriptional coactivator of p53 and co-immunoprecipitates with p53 from nuclear extracts [187,188]. RNA interference-induced suppression of p68 inhibits expression from DNA damage-induced p53 targets [187]. Ddx68 also co-immunoprecipitates with histone deacetylase 1 (HDAC), and can act as a transcriptional repressor of the herpes virus thymidine kinase promoter [189]. This protein forms a heterodimer with the RNA helicase Ddx2 (p72; 90% identity with p68), co-purifies with spliceosomes and acts at the U1 snRNA-5′ splice site duplex [190]. Reports show that p68 is important in the alternative splicing of the c-Ha-*ras* and *CD44* gene products and interacts with transcriptional coactivators CBP/p300 and RNA Pol II, which stimulates transcriptional activation mediated by CBP/p300 [191,192]. Buszczak *et al.* found that in *Drosophila*, p68 is required for clearance of transcripts from their synthesis sites, thus deactivating the relevant gene [193]. The authors suggest that as p68 was originally identified as a suppressor of position effect variegation in *Drosophila*, it may be involved in the formation of heterochromatin and RNAi, as the protein has been found in complex with Argonaut2 [193]. The isolation of p68 and other components of the spliceosome hint at a model where the deadbox protein may act as a coupling factor in a number of pathways, and is perhaps used by the virus at discrete steps in its life cycle. Would integrase interact with the spliceosome at the point of reverse transcription? In view of the growing number of splicing factors used by different viral proteins that have been identified by various groups, it seems apparent that there may be coordinated use of the same host factors by different viruses.

RanBP10 interacts with Ran and MET, a receptor-protein tyrosine kinase for hepatocyte growth factor (HGF) whose deregulation has been correlated with cancers in humans [194]. Ranbp10 shares 68% identity with RanBPM/RanBP9, but unlike RanBPM/RanBP9, RanBP10 does not interact with Sos, thus it is unable to activate the Ras/Erk pathway [194]. RanBP10 may thus function as a negative regulator for HGF via competition with RanBPM/RanBP9 for MET binding, or form alternative complexes with as yet undefined functions [194]. RanBP9 was identified in a yeast two-hybrid screen with acetylated HIV-1 IN (discussed above), and the isolation of two factors that may interact with one another by two different groups, with two different viral integrases is intriguing [102].

## 6. Perspectives and challenges

Moloney murine leukemia virus has been used in gene therapy trials, sometimes successfully, but sometimes with tragic outcomes. Two clinical gene therapy trials, one of which was conducted in France, used a replication defective MoMLV to transfer an interleukin receptor common gamma chain transgene to correct X-linked SCID disease (SCID-X1, severe combined immunodeficiency disease) in CD34^+^ cells [195]. Infants with this disease have no functional immune system and are subject to severe infections leading to mortality. Although the patients benefited from the gene therapy, the unfortunate outcome of the French trial was that three to six years after treatment, four of 10 children developed T cell leukemia and one child died [196]. In three of the patients this was due to the integration of the transgene at the *LMO2* locus (LIM domain only 2), a proto-oncogene normally expressed in hematopoetic cells, but not in mature T cells. The fourth patient had an integration into the BMI1 locus, another proto-oncogene [196]. The second study, conducted in the United Kingdom, used a similar MoMLV vector and resulted in one patient developing leukemia. This child also had an integration at the *LMO2* locus [197]. At least three of the children in the first and second studies also had additional contributing and complicating factors that included a translocation, deletions of cyclin-dependent kinase 2A (*CDKN2A*), and gain-of-function mutations in *NOTCH1* [196,197]. These loci are frequently mutated or deregulated in mouse cells containing MLV proviral insertions, so it should not be surprising that the same loci were affected in the gene therapy trials [198].

One mechanism of oncogene activation is transcriptional promotion from one of the viral LTRs. The second, most frequent mechanism is the activation of cellular promoters via enhancer elements in the LTRs. The third is by inactivation of negative regulatory elements in the oncogene or its transcript. The problems in the trials above appear to have originated with the enhancer sequences contained in the intact LTRs, emphasizing the need for a more complete understanding of the integration process for retroviruses if they are to be used as transgene delivery vehicles.

Understanding why MoMLV cannot infect non-dividing cells is critical for the expansion of its use in gene therapy. The ideal retroviral gene therapy vector would have the ability to transduce non-dividing cells; integrate at innocuous sites in the genome; provide long-term expression of the transgene; not be subjected to transcriptional silencing; and, not elicit a strong immunogenic response. This description suggests that HIV-1-derived vectors would satisfy these criteria, however, a major drawback to the use of this vector is that as a cytopathic virus, the effects of long-term expression are unknown. What too are the effects of long-term expression from a viral transgene that integrates within transcriptional units? The activity and effects of its accessory proteins on the long-term stability of transgenes in vectors with or without these factors (Rev, Tat, Nef, Vpr, and Vif) must be evaluated in detail. In addition, public fear of HIV-1 presents a significant barrier to its use in gene therapy, and these concerns should not be minimized. These questions must be addressed with respect to HIV-1 as a potential delivery vehicle.

The results of recent protein-protein interaction trap screens in yeast and *in vitro* binding assays suggest that there may be some common host proteins used by both the MoMLV and HIV-1 viruses [112]. If, indeed, this is borne out by functional tests, it would be unusual, as thus far, none of the HIV-1 integrase cofactors identified by other groups interact in the same manner with MoMLV integrase. The cDNA libraries screened with MoMLV IN were murine, so the question remains open as to whether or not all, if any of the clones isolated will ultimately exhibit equal effects on both HIV and MLV integration *in vitro*, or on virus infectivity *in vivo* [112].

With respect to improving the efficacy of MoMLV as a transgene delivery vehicle, ideal solutions would be to remove or modify sequences that prevent its nuclear entry in non-dividing cells and to determine the factors indigenous to the virus or to the host that are responsible for targeting, and to redirect integration to innocuous sites within the genome. Although no host proteins with PIC import or nuclear targeting activities analogous to those ascribed to LEDGF and those proposed for TRN-SR2 for HIV-1 have yet been verified with respect to MoMLV, a search for these factors should advance the development of safer MoMLV retroviral therapy vectors. Their identification and characterization will increase our understanding of the process of integration and may provide tools with which to manipulate the virus. The identification of host factors interacting with the MLV integrase protein suggests that the viral protein interacts with the host cell at a variety of steps and in multiple pathways. Perhaps, with the identification of cofactors, efforts to uncover the mechanism for integration site selection and nuclear entry for this virus will be facilitated.

## Figures and Tables

**Figure 1.**
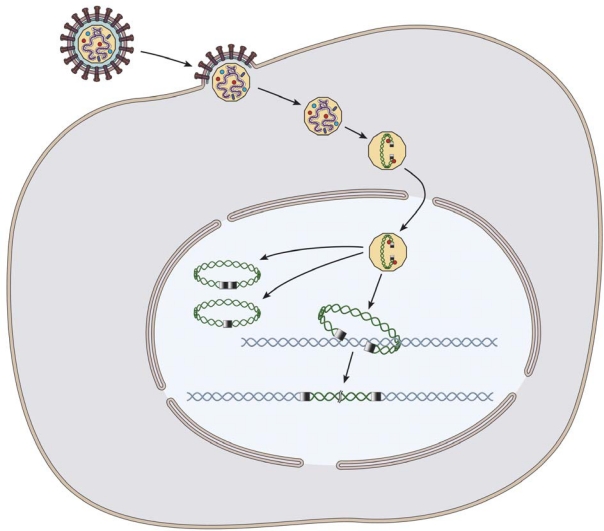
Early events in MLV infection leading to the production of the integrated provirus. Virions deliver a viral core particle into the cytoplasm, which carries out the process of reverse transcription to form the Pre-Integration Complex or PIC, containing the Integrase enzyme (red) bound to the termini of the Long Terminal Repeats or LTRs (grey boxes) of the viral DNA (green helix). The PIC enters the nucleus, likely via the dispersion of the nuclear membrane during mitosis. The incoming DNA is then integrated into the host genome (blue helix). DNAs that fail to integrate are often circularized by host repair enzymes to form circular DNAs with one or two copies of the viral LTRs.

**Table 1.** A compilation of selected host gene products identified as interacting with the MLV Integrase.

**Host Factors implicated in MLV PIC or IN interactions**	**Cellular Function**	**Proposed Function in viral life cycle**
Emerin, BAF, Lap2α.	Nuclear envelope; stabilization of actin cortical network	PIC importation, chromatin targeting of PIC; BAF inhibits autointegration of viral cDNA
Transcription factor IIE, beta subunit (TFIIE-β)	Subunit of RNA Pol II holoenzyme; recruits TFIIH to the Pol II-TFIIB-TFIID complex; stimulates RNA Pol II kinase and TFIIH DNA-dependent ATPase activities	Unknown; other subunits of basal Pol II complex isolated in other screens
Enhancer of zeste homolog 1 (Enx-1/Ezh2)	Polycomb Group 2 subunit complex with Eed and Suz12; chromatin structure maintenance and transcriptional regulation; Histone methyltransferase (H3K27 and H1K26)	Eed interacts with HIV-1 IN, Nef, and MA. Activity unknown in MLV
Flap endonuclease-1 (Fen1)	Removes 5′ initiator tRNA from Okazaki fragments; DNA repair in NHEJ and V(D)J recombination; 5′ to 3′ exonuclease and RNase H actvities	Resolution of free 5′ PO_4_ ends of viral DNA?
Ku70/XRCC6	NHEJ, chromosome and telomere maintenance, 70 kD subunit of Ku86 heterodimer, with Ku80 subunit of DNA-PKcs	Repair of gaps generated by IN cleavage at host/viral DNA junctions?
Tata binding protein ABT1 (ABT1)	Associates with Tata binding protein and activates basal transcription of class II promoters	Unknown
B-Activating transcription factor (B-ATF)	AP-1/ATF superfamily; Basic Leucine zipper transcription factor; blocks transformation by H-Ras and v-Fos; negative regulator of AP-1 mediated transcription by binding to Jun proteins	Unknown
All1 fused translocated to Chromosome 9 (AF9)/mixed lineage-leukemia translocated to 3 (Mllt3)	H3 hypermethylation; contains one YEATS domain (YNL107w/ ENL/AF-9/ and TFIIF small subunit); interacts with BCOR and MPc3 (Polycomb 3 homolog, component of PRC1)	Unknown
Bromodomain containing protein 2 (Brd2/Fsrg-1/RING3)	Bromodomain repeat-containing protein; mitogen-activated kinase activity; homolog of *Drosophila* female sterile homeotic gene; homodimer; interacts with histone H4 acetylated at lysine 13	Brd2 interacts with Latency-associated nuclear antigen (LANA-1) of KHSV
Zinc finger p38 (Znfp38)	Strong transcriptional activator; transactivation via its SCAN domain	Unknown
Peroxisome proliferative activated receptor, gamma, coactivator-1 related (PRC)	Serum-inducible coactivator of nuclear respiratory factor 1-dependent transcription from RNA pol II promoters; interacts with CREB1; stress response protein	Unknown
Ankyrin rep domain 49 (Ankrd49)	Putative transcription factor; contains acidic activation domain; Ankyrin repeat domain is similar to that of SWI6	Unknown
Splicing factor 3b, subunit 2 (SF3b2)	Putative DNA-binding (bihelical) motif predicted to be involved in chromosomal organization; SAP domain; basic domain in HLH proteins of MYOD family; component of spliceosome C complex; phosphorylated by ATM or ATR in response to DNA damage	Interacts with HIV-1 Vpr; identified in two or more studies
Splicing factor 3a, subunit 3 (SF3a3)	C2H2-type Zinc finger; mRNA processing; component of SF3A; associates with SF3B and 12S RNA unit to form U2 snRNP complex	Unknown
U2 auxiliary factor 26 (U2AF^26^)	Pre-RNA splicing factor; can replace U2AF1 in constitutive and enhancer dependent splicing activities; can replace U2AF^35^*in vitro*; enhances U2AF2 binding to weak Pyrimidine tracts.	Unknown
U5 small nuclear ribonucleoprotein (U5 snRNP)	Transcriptional regulation; SNF2 N-terminal domain; GTP binding factor; ortholog of *S. cerevisiae* splicing factor Prp8p; component of spliceosome C complex; interacts with Ddx5	Unknown
Step II Splicing factor SLU7	Pre mRNA splicing; required for 3′ splice-site choice by association with the spliceosome prior to recognition of the splice site in step II	Unknown
Survival of motor neuron (SMN)	Essential role in snRNP assembly; component of import snRNP complex containing Gemins 2, 3, 4, 5, 6 and 7; contains one Tudor domain; deficiency leads to apoptosis	Gemin 2 interacts with HIV-1 IN
Dead box p68 (Ddx68/Ddx5)	Component of spliceosome C complex; RNA-dependent helicase and ATPase activity; stimulated by ss-RNA; interacts with HDAC1	Identified in two or more genome wide studies (Bushman *et al*. 2009)
Ran binding protein 10 (RanBP10)	Competes with RanBP9 for MET binding; interacts with MET via its SPRY domain; interacts with Ran *in vitro*; does not interact with Sos nor activate Ras pathway	Several Ran family members identified in various studies (RanBP2, RanBP17); RanBP9 interacts with phosphorylated HIV-1 IN
Radixin	Member of ezrin, radixin, moesin family of actin binding proteins. Binds directly to barbed ends of actin filaments in plasma membrane.	ERM family member Moesin implicated in MLV and HIV viral trafficking
